# Determination of per- and polyfluoroalkyl substances in air samples from urban areas close to industrial complexes and human risk assessment

**DOI:** 10.1007/s11356-026-37431-6

**Published:** 2026-01-25

**Authors:** Reyes García-Garcinuño, Massimo Picardo, Josepa Fabregas, Laura Vallecillos, Francesc Borrull, Rosa Maria Marcé

**Affiliations:** 1 Department of Analytical Chemistry and Organic Chemistry, Universitat Rovira i Virgili, Campus Sescelades, Marcel·lí Domingo, 1, Tarragona, 43007 Spain; 2 Consorci d’Aigües Tarragona, Ctra. Nacional 340, Km.1094, L’Ampolla, Tarragona, 43895 Spain

**Keywords:** Per- and polyfluoroalkyl substances, Air particulate matter, Industrial areas, Risk assessment

## Abstract

An analytical method based on PM_10_ active sampling followed by pressurized liquid extraction and liquid chromatography-tandem mass spectrometry was developed to determine 20 per- and polyfluoroalkyl substances (PFASs). The method had good quality parameters, with method detection limits between 0.01 and 0.30 pg m^−3^, method quantification limits between 0.57 and 1.8 pg m^−3^, and recoveries above 75% for all target compounds. Analysis of air samples from two urban locations near industrial zones in Tarragona (Spain) revealed the widespread occurrence of PFASs, with perfluoro-n-butanoic acid (PFBA), perfluoro-n-pentanoic acid (PFPA), and sodium perfluoro-1-octanesulfonate (PFOS) as the most prevalent compounds at both sites. These three compounds were detected in all the samples analyzed, with the highest concentrations being 202 pg m^−3^ for PFBA, 178 pg m^−3^ for PFPA, and 109 pg m^−3^ for PFOS. Estimated daily intakes were calculated for infants, children, and adults under two scenarios. Risk assessment results showed individual non-carcinogenic risk values ranging from 2.4E-08 for perfluoro-n-undecanoic acid (PFUnDA) to 3.8E-01 for perfluoro-n-decanoic acid (PFDA), with the sum of all values remaining below the benchmark of 1, indicating that the risk is low. The compounds contributing most to the non-carcinogenic risk were PFDA (66.6%), PFOS (28.2%), and perfluoro-n-octanoic acid (PFOA, 5.10%). The carcinogenic risk was assessed for PFOS and PFOA, with the combined values in the range of 1.2E-06 to 3.7E-05, indicating also a low risk.

**Supplementary Information:**

The online version contains supplementary material available at 10.1007/s11356-026-37431-6.

## Introduction

Poly- and perfluoroalkyl substances (PFASs) are a group of persistent organic pollutants that contain over 9000 chemicals (USEPA 2022). These substances are part of a larger family of fluorine-based compounds, which are further categorized into subgroups. The two primary subgroups are polyfluoroalkyl and perfluoroalkyl substances, which are differentiated by the extent to which hydrogen atoms in their molecular structure are replaced by fluorine. In polyfluoroalkyl substances, the hydrogen atoms are only partially substituted, whereas in perfluoroalkyl substances, they are fully replaced. Some polyfluoroalkyl substances, such as fluorotelomer alcohol and N-methyl perfluorooctane sulphonamide, whose functional groups are alcohols or sulfonamides respectively, are characterized by their higher volatility and are predominantly found in the gas phase of the air samples. Perfluoroalkyl substances, on the other hand, include a subgroup known as perfluoroalkyl acids (PFAAs), which is further divided into two main categories: perfluorosulfonic acids (PFSAs) with sulfonate groups and perfluorocarboxylic acids (PFCAs) with carboxyl groups (Teymourian et al. 2021). This subgroup includes some of the most commonly detected PFASs, such as perfluoro-n-octanoic acid (PFOA), perfluoro-1-octane sulfonate (PFOS), and perfluoro-n-hexanoic acid (PFHxS). These compounds are less volatile than their polyfluoroalkyl counterparts and are therefore more commonly associated with the particulate phase of the air samples.

PFASs are found across various industrial and consumer products due to their remarkable chemical stability, which is attributed to strong carbon-fluorine bonds. Their widespread use has resulted in significant environmental contamination. PFASs are persistent, bioaccumulative, and resistant to environmental degradation, with long half-lives. They are therefore detected in water, sediment, air, plants, animal tissues, and even in remote regions, such as Arctic surface snow, which underscores their global transport potential (So et al. 2007; Young et al. 2007; Domingo et al. 2012; Teymourian et al. 2021; Chokwe et al. 2024). Mostly in water, PFASs have gained attention as hazardous substances under various international regulatory frameworks. In the European Union, some PFASs have been classified as priority substances under the Water Framework Directive due to their ecotoxicological impacts and the risks they pose to water resources (ED 2012). Additionally, they have been included in the control list under the Stockholm Convention on Persistent Organic Pollutants, which reflects their environmental and health hazards (Lallas 2001).

Although the EU has implemented restrictions on PFASs, these substances have still resulted in extensive environmental contamination and human exposure through pathways, such as ingestion, inhalation, and dermal contact (Wallington et al. 2006; Paul et al. 2009; Wong et al. 2018; ED 2020). Of particular concern is the growing presence of PFASs in the atmosphere, as their capability for long-range atmospheric transport significantly influences their environmental distribution and behavior (Ahrens et al. 2012; Yao et al. 2017; Teymourian et al. 2021).

The presence of PFASs in the air poses significant health risks due to their ability to disrupt lung surfactant function and cause acute toxicity upon inhalation. Ultrafine and submicron particles carrying PFASs can penetrate deep into the lungs, entering the bloodstream and contributing to systemic effects (Li et al. 2025). Prolonged exposure to these compounds has been linked to severe health issues, including cancer, thyroid hormone disruption, liver toxicity, chronic kidney disease, and reproductive disorders in both men and women (Teymourian et al. 2021). PFAS exposure can also lead to developmental issues, such as reduced birth weight and precocious puberty (Teymourian et al. 2021). Additionally, the widespread environmental presence of PFASs highlights the urgent need for further research into their health impacts and mitigation strategies (Fromme et al. 2009; Zhao et al. 2012).

To determine PFAS in air samples, the most volatile PFASs, which are usually found in the gas phase, are retained using polyurethane foam (PUF) samplers (Karásková et al. 2018; Wu et al. 2021) or by thermal desorption with sorbent tubes (Wu and Chang 2012). Less volatile compounds are typically associated with particulate matter such as PM_10_, and to collect PM_10_ samples, quartz fiber filters (QFFs) are commonly employed (Zhang et al. 2016; Yu et al. 2018; Liu et al. 2019). Glass fiber filters (GFF), which are also commonly used for PM_10_ sampling, are not suitable for PFASs analysis, as Johansson et al. (2017) reported that they exhibit significant sorption of gaseous PFOA at low atmospheric concentrations. These filters retain PFASs that are later extracted using techniques such as ultrasound extraction and centrifugation (Yu et al. 2018; Liu et al. 2019; Li et al. 2025), ultrasonic agitation (Kourtchev et al. 2023), Soxhlet extraction (Androulakakis et al. 2022), and pressurized liquid extraction (PLE) (Wong et al. 2018). Once extracted, these compounds are often determined by gas chromatography-mass spectrometry (GC-MS) for the more volatile components (Wu and Chang 2012; Wong et al. 2018) or by liquid chromatography mass spectrometry (LC-MS) and liquid chromatography tandem mass spectrometry (LC-MS/MS) for the others (Ahrens et al. 2013, 2012, 2011; Liu et al. 2015).

In previous studies, the main PFASs compounds found in particulate matter were perfluoro-n-butanoic acid (PFBA), perfluoro-n-pentanoic acid (PFPeA), and PFOA by Zhang et al. (2016) and perfluoro-n-hexanoic acid (PFHxA) and perfluoro-tetradecanoic acid by Li et al. (2025). The distribution and concentration of PFASs in particulate matter are influenced by urban activities, including household combustion (e.g., cooking emissions), diurnal patterns of road traffic, industrial activity, and the proximity of industrial parks to residential areas (Cai et al. 2018; Xu et al. 2019). For example, 21 PFASs were found in PM_2.5_ in industrial and urban areas of Curitiba (Brazil), with concentrations ranging from 9.62 pg m^−3^ for perfluoro-n-decanoic acid (PFDA) to 136 pg m^−3^ for PFHxA (Li et al. 2025). In PM_10_, 17 PFASs were found at an annual average concentration between 23.6 and 94.5 pg m^−3^ in cities along the Bohai and Yellow Seas coasts in northeastern China (Yu et al. 2018). Furthermore, studies in North Carolina (USA) show that legacy PFASs (e.g., PFOS and PFOA) are regionally distributed, whereas emerging PFASs exhibit higher concentrations near point sources such as fluoropolymer manufacturing facilities. This pattern has been observed in PM_2.5_ samples from multiple urban sites (Zhou et al. 2021) and confirmed by atmospheric monitoring around the Chemours Fayetteville Works plant (North Carolina, USA) (Zhou et al. 2022), highlighting the stronger association of emerging PFASs with local emissions.

To better understand the presence and health risks associated with PFASs, the aims of this study are (1) to develop and validate a multi-residue method based on PLE followed by LC-MS/MS to determine up to 20 PFASs in PM_10_ air samples, (2) monitor the presence of these compounds in particulate matter outdoor air samples from urban locations close to industrial activities, and (3) evaluate the non-carcinogenic and carcinogenic risks of these compounds for different age groups (infants, children, and adults) through outdoor air inhalation in two exposure scenarios.

## Experimental part

### Reagents and standards

HPLC grade solvents with purities < 99.9%, such as water, methanol (MeOH), acetonitrile (ACN), ammonium acetate, and acetone were obtained from Carlo Erba (Cornaredo, Milan, Italy). Nitrogen gas for PLE, with a purity of 99.999%, was from Carburos Metálicos (Tarragona, Spain). The standard mixture consists of the following 20 PFASs at a concentration of 2,000 µg L^−1^ in methanol (Wellington Laboratories, Whitby, ON, Canada): PFBA, PFPeA, PFHxA, PFHpA, PFOA, PFNA, PFDA, PFUnDA, PFDoA, PFTrDA, PFBS, PFPeS, PFHxS, PFHpS, PFOS, PFNS, PFDS, PFUdS, PFDoS, and PFTrDS. A mixture of the following 13 isotopically labelled PFAS compounds at a concentration of 2000 µg L^−1^ (Wellington Laboratories) was used as internal standards (IS): ^13^C PFBA, ^13^C PFPeA, ^13^C PFHxA, ^13^C PFHpA, ^13^C PFOA, ^13^C PFNA, ^13^C PFDA, ^13^C PFUdA, ^13^C PFDoA, ^13^C PFTeDA, ^13^C PFBS, ^13^C PFHxS, and ^13^C PFOS. Two working solutions of 500 µg L^−1^ of PFASs and IS were prepared in methanol (MeOH) and stored in amber glass vials at −20 °C. The full list of target PFASs evaluated, along with the IS used and their respective acronyms, is provided in Table 1S.

### Sampling

**Fig. 1:**
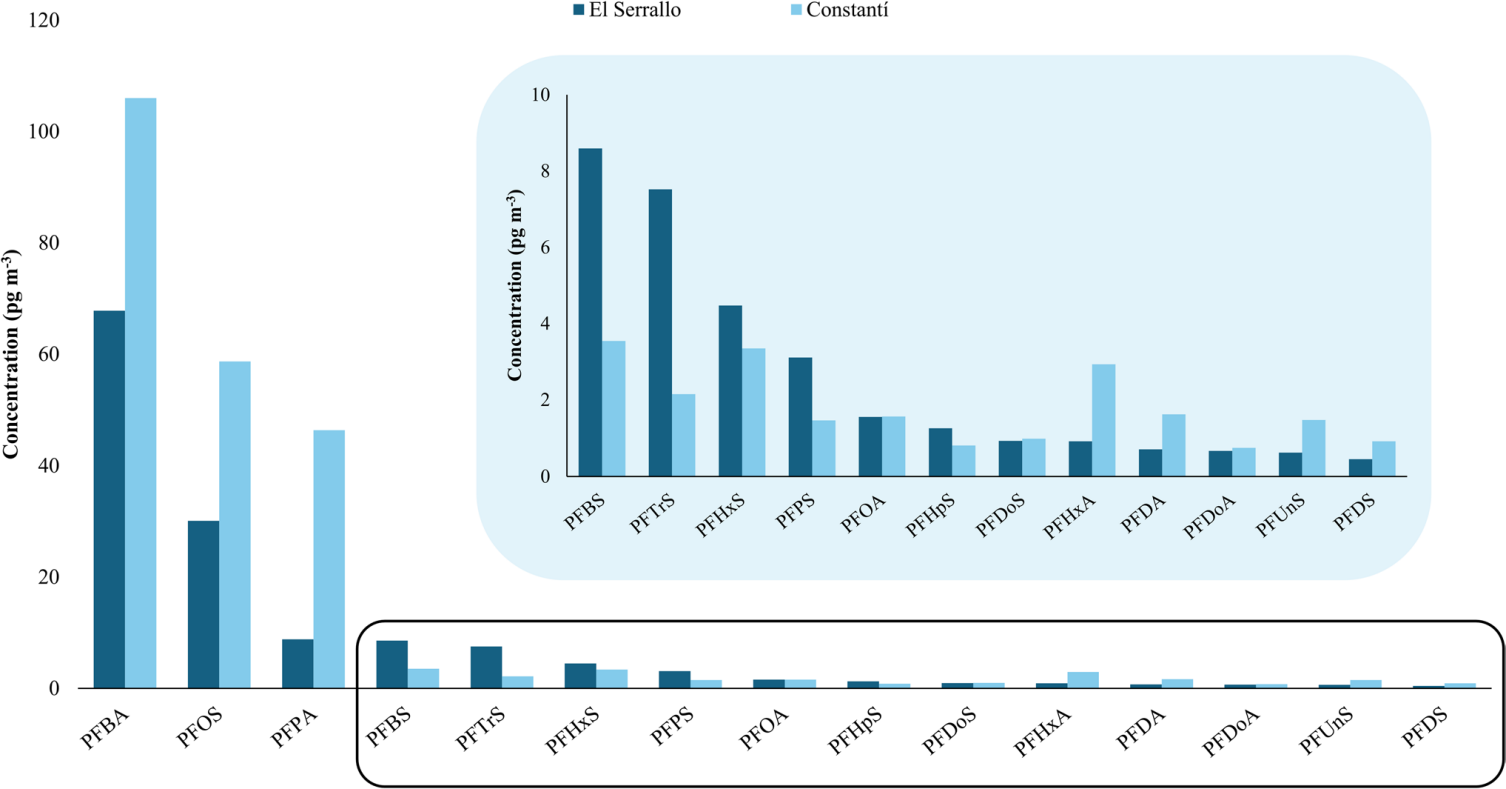
Bar graph showing the concentrations of the target PFASs at each sampling site

Sampling took place at two stations of the Network for Monitoring and Forecasting Air Quality of the Catalan Government, located in the Tarragona area. These stations were located taking into account the prevailing winds in the region, and there are no surrounding buildings or trees that could cause shielding effects. One of the sampling sites was in Constantí (41.155011, 1.217653), a small town of about 7000 inhabitants located 3 km away from the northern industrial park of Tarragona. The other site was located in El Serrallo (41.109836, 1.243226), the neighborhood closest to the port of Tarragona and about 3 km from the city’s southern industrial park. Figure 1S shows a map with the sampling sites and the locations of the two industrial parks, the port of Tarragona and Reus airport. The northern park contains an oil refinery and chemical industries, including benzene and 1,3-butadiene production plants, while the southern park specializes in producing plastics (e.g., PVC, ABS) and chlorinated compounds (e.g., 1,2-dichloroethane). The Port of Tarragona, with chemical handling facilities and a small refinery, exports over half of the city’s petrochemical production (AEQT 2025). Reus Airport is exclusively for passenger traffic, with peak activity from early April to late October. During the sampling period, the number of passengers ranged from 996 to 4176 (AENA 2025).

Ten PM_10_ samples from each site were collected simultaneously at both locations between November 2023 and January 2024. Sampling took place during thermal inversion events under the prevailing north (N) and north-westerly (NW) winds typical of the region in winter. Regardless of the sampling site, temperatures ranged from 2.80 to 21.2 °C, with relative humidity percentages between 28 and 96% (METEO 2025). Samples were collected using an MCV-PM10 High Volume Air Sampler, manufactured by MCV S.A. (Collbató, Barcelona). Particulate matter of ≤ 10 µm (PM_10_) was deposited on conditioned 150-mm-diameter quartz fiber filters (QFF) from Whatman (Sigma Aldrich, St. Louis, USA), and 24-h samplings were performed at a constant flow rate of 30 m^3^ h^−1^ for a total volume of roughly 720 m^3^. The QFF samples were carefully wrapped in aluminum foil, labelled, and stored in a freezer at −20 °C until they were analyzed.

### QFFs extraction

The PLE of QFFs was carried out in an ASE 350 Accelerated Solvent Extraction system manufactured by Dionex (Sunnyvale, CA, USA), and stainless-steel cells of 10 mL were used. To set up the extraction, a cellulose filter (Thermo Scientific, Barcelona, Spain) was set at the bottom of the cell, followed by half of a QFF cut with scissors and diatomaceous earth (Thermo Scientific) until the cell was full. The scissors had been previously cleaned with acetone, and the diatomaceous earth has been conditioned overnight at 400 °C. MeOH was used as the solvent for the extraction process, and the temperature was set at 100 °C and pressure set at 1500 psi. The time for preheating and static extraction was both 5 min. The flushing volume was 50% of the volume of the extraction cell, and the nitrogen purge was 120 s.

Fifty microliters of a mixed solution of the internal standards of 500 µg L^−1^ was added to the PLE extract. Then, they were evaporated to dryness with a miVac Duo centrifuge evaporator by Genevac (Ipswich, UK). The dried residues were subsequently reconstituted with 500 µL of ACN, filtered through a 0.45-µm regenerated cellulose syringe filter from LLG Labware (Meckenheim, Germany), and adjusted to a final volume of 1 mL with H_2_O (10% acetic acid). Fifty microliters of this solution was injected into the LC-MS/MS system.

### Chromatographic conditions

The LC-MS/MS analyses were conducted using a chromatographic system from Agilent Technologies (Waldbronn, Germany) equipped with a 1260 binary pump, 1260 autosampler, 1260 HiP degassing unit, 1260 thermostated column compartment, and the triple quadrupole analyser. Briefly, a Poroshell 120 EC-C18 (50 × 3 mm, 2.7 µm) chromatographic column and a guard column (120 EC-C18; 5 × 3 mm, 2.7 µm) were used. The mobile phase consisted of HPLC grade water with 10 mM ammonium acetate (A) and ACN (B). The gradient profile initiated at 10% B, increased to 40% B within 2 min, further increased to 95% B in 4 min, and reached 100% B in 6.5 min. Finally, it returned to the initial conditions (10% B) within 0.5 min and was held steady for 5 min to equilibrate the column for subsequent analyses. A constant flow rate of 0.40 mL min^−1^ was applied, and the column was set at 45 °C. The injection volume was 50 µL. The acquisition was conducted in dynamic multiple reaction monitoring (diMRM) in negative mode utilizing an electrospray ionization (ESI) source. The optimal conditions for the ESI included a nitrogen flow rate of 8 mL min^−1^, a nebulizer pressure of 30 psi (N_2_), and a source temperature of 300 °C. Collision energies for all compounds ranged from 4 to 200 eV. One transition for quantification and another for qualification were used. Table 2S shows the retention times and MRM mass transitions for the 20 target PFASs and the 13 isotopically labelled PFASs. More detailed information regarding the chromatographic method can be found in Martínez et al. (2024).

Internal standard calibration curves showed good linearity up to 250 µg L^−1^ for all target compounds with determination coefficients (*R*^2^) greater than 0.990 in all cases. The instrument limits of detection (ILODs) (Table 2S), set as the concentrations with a signal-to-noise ratio equal to or higher than three, were in the 0.07–0.20 µg L^−1^ range. The instrument limits of quantification (ILOQs), which were defined as the lowest concentration in the calibration curves, were between 0.50 and 1.0 µg L^−1^.

### Quality assurance/quality control

To minimize background contamination, an analytical LC column (Poroshell 120 EC-C18 (100 × 4.6 mm × 2.7 µm)) was positioned as a delay column, situated between the mobile phase mixing chamber and the sample injector. Additionally, all polyetheretherketone tubes (PEEK) were replaced with stainless steel counterparts. The extractions were performed with material previously washed with isopropanol to prevent cross-contamination. The QFFs were conditioned at 400 °C for 24 h and, until use, were covered with aluminum foils and kept in the freezer at −20 °C. The blank QFFs were analyzed for the presence of the studied compounds, but none was found at quantifiable levels.

For quality assurance purposes, instrumental and procedural blanks were performed periodically. To ensure correct behavior of the instrument, standard controls of 25 µg L^−1^ were also included in the LC-MS/MS batches.

### Risk assessment

The concentrations of the target compounds found in PM_10_ samples were used to calculate the estimated daily intake (EDI, pg kg_bw_^−1^ day^−1^) for ambient inhalation, employing Eq. 1 (Asante-Duah 2002):

1 $$\mathrm{EDI}=\frac{\mathrm{C}\times\mathrm{IR}\times\mathrm{RR}\times\mathrm{ET}\times\mathrm{EF}\times\mathrm{ED}}{\mathrm{BW}\times\mathrm{AT}}$$

The equation includes parameters such as the concentration of compounds in PM_10_ (C, pg m^−3^), inhalation rates (IR, m^3^ h^−1^), and retention rate of inhaled air (%). Additionally, exposure time (ET, h day^−1^), exposure frequency (EF, days years^−1^), and exposure duration (ED, years) were factored against body weight (BW, kg) and average exposure periods (AT, days). Parameter values, detailed in Table 3S (Asante-Duah 2002), enable EDI calculations across scenarios (e.g., high vs. low) and age groups (infants, children, and adults). Furthermore, to evaluate the non-carcinogenic risk of some compounds, the levels defined as reference dose (RfD) by the USEPA Risk Assessment Integration System (RAIS 2025) were used to estimate non-cancer risk by calculating hazard quotients (HQs) using Eq. 2. The RfD values are shown in Table 4S.

2 $$HQ=\frac{EDI}{RfD}$$

To estimate the carcinogenic risk (CR), Eq. 3 was applied. The oral slope factor (SFo) values are shown in Table 4S (RAIS 2025).

3 $$CR=EDI\times SFo$$

## Results

Although the LC-MS/MS method was previously developed (Martínez et al. 2024), quality parameters of the instrumental method were determined and values are shown in the Sect. “Quality assurance/quality control.” This LC-MS/MS method was used for PLE optimization, and once the optimal conditions were obtained, the global method was validated as will be described in Sect. “Method validation.” 

### PLE optimization

Initial PLE conditions were set according to previous experience in the extraction of semi-volatile organic compounds from QFF samples (Maceira et al. 2019; Garcia-Garcinuño et al. 2024). Due to the polarity of PFASs, ACN and MeOH were tested as extraction solvents, and different temperatures, at 70 °C and 100 °C, were tested. The other PLE conditions were 5 min for the time of preheating and static extraction, 1 cycle, a flush volume of 50%, and 120 s of purge time. To conduct the optimization, previously conditioned QFFs were divided into two halves, one of which was spiked with 50 µL of a standard solution of 500 µg L^−1^ of all target compounds, and the other was used to subtract possible compounds still present in the conditioned QFF blanks. All conditions were evaluated in triplicate (25 ng, *n* = 3).

The recoveries obtained with the different conditions were very similar as can be seen in Table 5S. Finally, the optimal conditions for extraction were with MeOH as extraction solvent and a temperature of 100 °C, as the evaporation step was faster and allowed for the analysis of more samples per day. The recoveries obtained in this study, which were between 76 and 113%, were similar to those reported in other studies focused on the determination of PFASs in outdoor air samples. For instance, ultrasound-assisted extraction with MeOH provided recoveries between 82 and 114% (Guo et al. 2018) and between 93.8 and 122% for 21 PFASs (Li et al. 2025), while mechanical shaking and vortex mixing with MeOH resulted in recoveries between 65 and 96% (Lin et al. 2020). Although, to the best of our knowledge, PLE has not previously been applied for the extraction of target PFASs retained in QFFs (particulate matter), similar recoveries (65 to 109%) were achieved when using PLE to extract the most volatile PFASs in polyurethane foams (gaseous phase) (Wong et al. 2018).

### Method validation

The developed PLE/LC-MS/MS method was validated using sampled QFFs to evaluate recoveries, matrix effect (ME), method detection limits (MDLs), method quantification limits (MQLs), repeatability, and reproducibility between days. To calculate the recoveries, the procedure described in Sect. “QFFs extraction” was applied to sampled QFF from Constantí. The recoveries obtained at two concentration levels (10 ng and 25 ng) are summarized in Table 1. Regardless of the concentrations added to the sampled QFFs, recovery values ranged from 71% for PFDoS to 121% for PFUnDs. To perform the ME test, 25 ng of the target compounds and the ISs were added to the extracts of the sampled QFFs and the volume was made up to 1 mL. As Table 1 shows, the ME values ranged from −26% for PFDoS to 9% for PFTrA. This confirmed that even for the target PFASs without a deuterated equivalent, the ISs used minimized the ME. The recovery values obtained for both the blank and sampled QFFs were quite similar. Therefore, target PFASs quantification was performed using IS standard calibration, and recoveries were used to calculate the final sample concentration. MDLs and MQLs were estimated from the ILODs and ILOQs for each target compound, using recoveries and the sample volume (720 m^3^). As shown in Table 1, MDLs ranged from 0.09 pg m^−3^ for PFPA, PFDoA, and PFDA to 0.30 pg m^−3^ for FPHpS. MQLs ranged from 0.60 pg m⁻^3^ for PFDoA and PFUnS to 1.8 pg m^−3^ for PFPA. Method repeatability (25 ng, *n* = *3*), expressed as a percentage of relative standard deviation (%RSD), was below 15%, and method reproducibility (25 ng, *n* = *3*) was below 18%.

**Table 1:** Method detection limits (MDLs) and method quantification limits (MQLs) expressed in pg m^−3^, recoveries (%) and their repeatability in brackets (RSD%, *n* = 3) for blank and sampled QFFs and the matrix effect for each target PFASs

Compounds	MDL (pg m^−3^)	MQL (pg m^−3^)	Recovery (*n* = 3)			Matrix effect (*n* = 3)
			Blank QFF	Sampled QFF		
			25 ng	25 ng	10 ng	25 ng
PFBA	0.20	0.80	84 (7)	90 (6)	100 (8)	−3
PFOS	0.10	0.80	80 (9)	82 (6)	92 (7)	−5
PFPA	0.09	1.8	91 (6)	87 (11)	98 (6)	−2
PFBS	0.10	0.80	87 (8)	83 (5)	93 (5)	−6
PFTrS	0.30	1.3	113 (5)	108 (8)	120 (4)	−2
PFHxS	0.10	0.80	87 (7)	86 (6)	96 (7)	−5
PFPS	0.20	1.0	79 (6)	80 (5)	88 (15)	−10
PFOA	0.20	0.90	96 (4)	87 (3)	102 (9)	7
PFHpS	0.30	0.80	80 (9)	79 (6)	88 (10)	−15
PFDoS	0.20	0.90	76 (5)	79 (11)	71 (11)	−26
PFHxA	0.30	0.80	94 (4)	84 (10)	93 (6)	−6
PFDA	0.10	0.70	88 (5)	102 (6)	104 (4)	2
PFDoA	0.09	0.60	94 (7)	121 (5)	118 (4)	13
PFUnS	0.10	0.60	90 (9)	121 (4)	100 (8)	−13
PFDS	0.10	0.90	85 (8)	81 (5)	105 (5)	−18
PFNS	0.20	1.0	77 (11)	73 (12)	86 (6)	−10
PFTrA	0.10	0.80	94 (2)	91 (10)	107 (4)	9
PFUnDA	0.10	0.80	83 (9)	88 (10)	96 (5)	−4
PFHpA	0.30	0.90	102 (5)	82 (10)	91 (5)	−9
PFNA	0.20	1.0	87 (6)	89 (7)	99 (5)	−2

### Occurrence in particulate matter from air samples

The sum of the 20 PFASs studied (∑PFASs) ranged from 4.15 to 527 pg m^−3^, with average values of 139 pg m^−3^ in El Serrallo and 234 pg m^−3^ in Constantí. All target PFASs were detected in at least one of the samples analyzed. PFBA, PFPA, and PFHxA were found in all samples (100% detection rate). PFNA was detected but always at concentrations below the MQL. Table 2 summarizes the average concentrations, the concentration range, and the detection rate observed for both sites. Regardless of the sampling site, Fig. 1 shows the compounds found at the highest concentration were PFBA, PFOS, and PFPA. The concentrations of these compounds ranged from < MDL to 202 pg m^−3^ for PFBA, from < MDL to 178 pg m^−3^ for PFPA, and from < MDL to 109 pg m^−3^ for PFOS. Although these compounds had a 100% detection rate at both sampling sites, the average concentrations found in Constantí (106 pg m^−3^ for PFBA, 46.4 pg m^−3^ for PFPA, and 58.7 pg m^−3^ for PFOS) were higher than those found in El Serrallo (67.8 pg m^−3^ for PFBA, 8.59 pg m^−3^ for PFPA, and 30.1 pg m^−3^ for PFOS). This is likely because the prevailing winds during sampling were from the N and NW, so Constantí, in addition to receiving PFAS emissions from urban activities, was also influenced by emissions from the northern industrial park of Tarragona. Yu et al. (2018) also identified PFBA and PFOS as the most prevalent compounds in PM_10_ samples from the city centers of urban areas along the coastal regions of the Bohai and Yellow Seas (Northeast China), which are characterized by the presence of PFAS manufacturers. Concentrations ranged from 5.2 to 25.9 pg m^−3^ for PFBA and from 0.33 to 26 pg m^−3^ PFOS. PM_10_ samples from Peking University, a site representative of a typical urban environment of Beijing, contained higher concentrations of PFPA, ranging from 43 to 830 pg m^−3^ (Wu et al. 2019). Although some sampling sites were located near electronic factories, machine-building and chemical plants, and the port connected to Hong Kong, Liu et al. (2015) reported lower average concentrations of PFBA (1.9 pg m^−3^), PFPA (1.9 pg m^−3^), and PFOS (4.3 pg m^−3^) in passive air samples from Shenzen, China. This is likely because the distance between the industrial areas and the urban sampling sites (10–30 km) was much greater than in the present study (3 km).

**Table 2:** Concentrations of PFASs (pg m^−3^) in PM_10_ samples collected in El Serrallo and Constantí

Compounds	El Serrallo			Constantí		
	Avg^a^	Range	DR^b^	Avg^a^	Range	DR^b^
PFBA	67.8	< MQL–164	100	106	< MQL–202	100
PFOS	30.1	< MQL–72.5	100	58.7	n.d.–109	90
PFPA	8.79	< MQL–61.2	100	46.4	< MQL–178	100
PFBS	8.59	< MQL–53.4	100	3.54	n.d.–13.2	90
PFTrS	7.52	< MQL–59.2	100	2.15	n.d.–8.85	80
PFHxS	4.47	n.d.–16.7	90	3.35	n.d.–7.73	80
PFPS	3.11	< MQL–14.5	100	1.47	n.d.–6.28	80
PFOA	1.56	< MQL–11.6	100	1.57	n.d.–4.70	70
PFHpS	1.26	n.d.–3.24	80	0.81	n.d.–2.29	70
PFDoS	0.93	n.d.–4.44	60	0.99	n.d–2.45	30
PFHxA	0.92	< MQL–3.82	100	2.93	< MQL–10.8	100
PFDA	0.71	n.d.–4.73	40	1.63	n.d.–3.11	90
PFDoA	0.67	n.d.–4.00	20	0.75	n.d.–1.71	90
PFUnS	0.62	n.d.–2.90	60	1.48	n.d.–8.85	90
PFDS	< MQL	n.d.–2.61	20	0.92	n.d.–3.61	50
PFNS	< MQL	n.d.–3.90	30	< MQL	n.d.–1.44	40
PFTrA	< MQL	n.d.–2.36	20	< MQL	n.d.–0.83	80
PFUnDA	< MQL	n.d.–1.96	20	< MQL	n.d.–0.87	90
PFHpA	< MQL	n.d.–3.85	70	< MQL	n.d.–0.74	40
PFNA	< MQL	n.d.–< MQL	70	< MQL	n.d.–< MQL	50
**∑PFASs**	139	6.28–346	-	234	4.15–527	-

^a^Average concentration

^b^Detection rate (DR)

A second group of compounds, including PFBS, PFHxS, and PFTrS, showed concentrations between < MQL and 59.2 pg m^−3^ regardless of the sampling site, with detection rates between 80 and 100%. In this case, the average concentrations of these three compounds were between 2.15 pg m^−3^ for PFTrS and 8.59 pg m^−3^ for PFBS, with slightly higher values found in El Serrallo. The average values found for PFHxS were also similar to those reported by Wu et al. (2019) in PM_10_ samples from Beijing (China) and by Paragot et al. (2020) in similar samples from the Czech Republic. On the other hand, compounds such as PFHxA, PFPS, PFOA, PFHpS, PFDA, PFUnS, and PFDoS showed average concentrations ranging from 0.62 to 3.11 pg m^−3^, with episodic values up to 14.5 pg m^−3^ for PFPS and 11.6 pg m^−3^ for PFOA, both in Constantí. All these compounds, except PFPS and PFHpS, were found at slightly higher average concentrations in Constantí, likely due to the lower detection ranges (40% and 100%) found in El Serrallo. Moreover, the average PFHxA concentrations found in El Serrallo (0.72 pg m^−3^) and Constanti (2.93 pg m^−3^) were similar to the 1.5 pg m^−3^ found in passive air samples from Shenzen by Liu et al. (2015). A lower average value of PFHxA of 0.27 pg m^−3^ was found in air samples from a non-industrialized area in the Czech Republic (Paragot et al. 2020).

On the other hand, the compounds with the lowest concentrations in this study were the following: PFHpA, PFNS, PFUnDA, PFDS, PFDoA, PFDoS, and PFTrA. Their average concentrations were between < MQL and 0.92 pg m^−3^, with the Constantí values being slightly higher in general. Quantifiable values were in the range of 0.74 pg m^−3^ and 4.00 pg m^−3^ with detection rate percentages mostly between 20 and 60%. The concentrations values found for PFUnDA and PFHpA in the present study were comparable to those reported in PM_10_ samples from Beijing (Wu et al. 2019) and the Czech Republic (Paragot et al. 2020), respectively. In contrast, Li et al. (2025) found higher concentrations of 14.9 pg m^−3^ and 16.3 pg m^−3^ for PFUnDA and PFTrDA, respectively. Some of these low-abundance PFASs have also been detected in PM_2.5_ samples from North Carolina (USA). Compounds such as PFHpA, PFUnDA, and other long-chain PFASs were observed at similarly low concentrations (< MQL–0.14 pg m^−3^). These findings further support that the trace-level occurrence of these PFASs is consistent across different regions and atmospheric particle size fractions.

### Human exposure and risk assessment

To assess human exposure to PFASs via inhalation, estimated daily intakes (EDIs) were calculated for both El Serrallo and Constantí under two exposure scenarios: a low-case scenario based on the geometric mean of observed concentrations in PM_10_ samples and a high-case scenario based on the 95th percentile of those concentrations. This evaluation included three age-based population groups: infants, children, and adults. Following USEPA guidelines, EDIs for target compounds detected at levels below the MDL and MQL were estimated by substituting concentration values with MDL/2 and MQL/2, respectively (USEPA 2007).

**Fig. 2:**
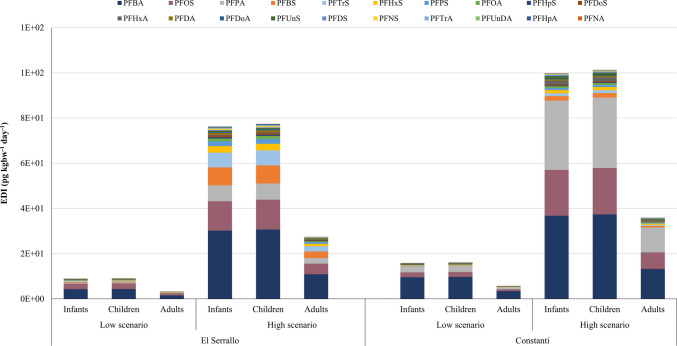
EDIs (pg kg_bw_^−1^ day^−1^) calculated for each compound in two different exposure scenarios, three population groups, and the two sampling sites

The sum of EDIs (∑EDIs) values were between 3.2 pg kg_bw_^−1^ day^−1^ (El Serrallo, low scenario, adults) and 101 pg kg_bw_^−1^ day^−1^ (Constantí, high scenario, infants and children). Figure 2 shows that the EDIs obtained for infants and children were higher than those for adults at both sampling sites, mainly due to their lower body weight. Overall, the EDIs in Constanti were slightly higher than those in El Serrallo due to the higher concentrations detected. As Table 6S shows, PFBA, PFPA, and PFOS had the highest EDI values, which ranged from 1.6E-01 pg kg_bw_^−1^ day^−1^ (PFPA, El Serrallo, low scenario, adults) to 3.7E + 01 pg kg_bw_^−1^ day^−1^ (PFBA, Constantí, high scenario, infants and children). The EDIs of the remaining PFASs were within the range of 1.4E-03 pg kg_bw_^−1^ day^−1^ (PFDoA, El Serrallo, low scenario, adults) to 8.0E + 00 pg kg_bw_^−1^ day^−1^ (PFBS, El Serrallo, high scenario, children). The EDIs obtained in the present study were higher than those reported by Liu et al. (2019), who found EDI values for PFOA up to 8.1E-01 pg kg_bw_^−1^ day^−1^ and for PFOS up to 4.5E-02 pg kg_bw_^−1^ day^−1^. Similarly, the study by Liu et al. (2015) reported EDIs for PFOA and PFOS of 4.8E-02 pg kg_bw_^−1^ day^−1^ and 2.8E-02 pg kg_bw_^−1^ day^−1^, respectively. These values are two orders of magnitude lower than those observed in Constantí and El Serrallo, due to the elevated PFAS concentrations found at our sampling sites.

The HQ risk assessment for each target compound, categorized by two scenarios (low and high) across three age groups (infants, children, and adults) in both El Serrallo and Constanti, is found in Table 3. HQ values ranged from 2.4E-08 (PFUnDA, El Serrallo, low scenario, adults) to 3.8E-01 (PFDA, El Serrallo, high scenario, infants and children). These values were obtained only for the ten PFAS listed in Table 3S, since they have the potential to cause adverse health effects at certain doses and have RfD values available (RAIS 2025).

**Table 3:** HQ and CR values for the target PFASs with risk values for two exposure scenarios and three population groups in El Serrallo and Constantí

Compounds		El Serrallo						Constantí					
		Low scenario			High scenario			Low scenario			High scenario		
		Infants	Children	Adults	Infants	Children	Adults	Infants	Children	Adults	Infants	Children	Adults
**HQ**	PFBA	4.3E-06	4.3E-06	1.5E-06	3.0E-05	3.1E-05	1.1E-05	9.6E-06	9.7E-06	3.4E-06	3.7E-05	3.7E-05	1.3E-05
	PFOS	2.5E-02	2.5E-02	8.9E-03	1.3E-01	1.3E-01	4.7E-02	2.2E-02	2.2E-02	7.8E-03	2.0E-01	2.1E-01	7.3E-02
	PFBS	1.0E-06	1.0E-06	3.6E-07	2.6E-05	2.7E-05	9.5E-06	4.4E-07	4.4E-07	1.6E-07	6.6E-06	6.7E-06	2.4E-06
	PFHxS	1.2E-05	1.2E-05	4.3E-06	1.4E-04	1.5E-04	5.2E-05	6.8E-06	6.9E-06	2.5E-06	7.1E-05	7.2E-05	2.6E-05
	PFOA	3.7E-03	3.7E-03	1.3E-03	4.1E-02	4.2E-02	1.5E-02	3.6E-03	3.7E-03	1.3E-03	2.6E-02	2.7E-02	9.4E-03
	PFHxA	1.9E-07	1.9E-07	6.8E-08	8.6E-07	8.7E-07	3.1E-07	5.2E-07	5.3E-07	1.9E-07	3.3E-06	3.3E-06	1.2E-06
	PFDA	5.8E-02	5.9E-02	2.1E-02	3.8E-01	3.8E-01	1.4E-01	7.8E-02	8.0E-02	2.8E-02	2.9E-01	2.9E-01	1.0E-01
	PFDoA	8.0E-08	8.2E-08	2.9E-08	8.7E-06	8.9E-06	3.2E-06	1.5E-06	1.5E-06	5.4E-07	6.0E-06	6.1E-06	2.2E-06
	PFUnDA	6.7E-08	6.8E-08	2.4E-08	7.8E-07	8.0E-07	2.8E-07	1.4E-07	1.4E-07	4.9E-08	5.2E-07	5.3E-07	1.9E-07
	PFNA	1.7E-05	1.3E-05	6.2E-06	3.0E-05	3.0E-05	1.1E-05	9.5E-06	9.7E-06	3.4E-06	3.0E-05	3.0E-05	1.1E-05
	**∑HQ**	8.7E-02	8.7E-02	3.1E-02	5.4E-01	5.5E-01	2.0E-01	1.0E-01	1.1E-0.1	3.7E-02	5.2E-01	5.3E-01	1.8E-01
**CR**	PFOS	9.7E-08	9.9E-08	3.5E-08	5.1E-07	5.2E-07	1.9E-07	8.6E-08	8.7E-08	3.1E-08	8.0E-07	8.1E-07	2.9E-07
	PFOA	3.2E-06	3.3E-06	1.2E-06	3.6E-05	3.7E-05	1.3E-05	3.2E-06	3.2E-06	1.1E-06	2.3E-05	2.3E-05	8.3E-06
	**∑CR**	3.3E-06	3.4E-06	1.2E-06	3.7E-05	3.7E-05	1.3E-05	3.3E-06	3.3E-06	1.2E-06	2.4E-05	2.4E-05	8.6E-06

**Figure. 3:**
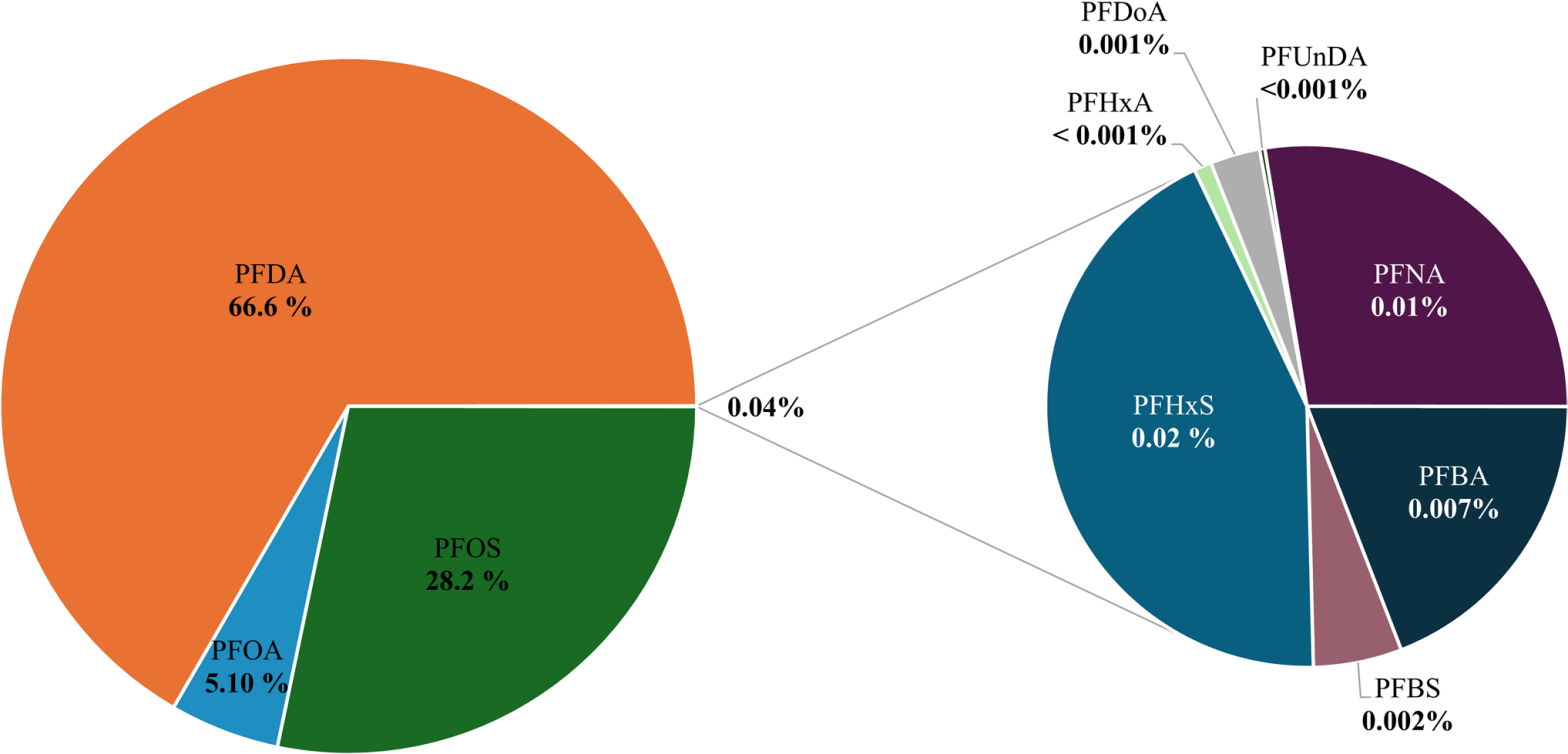
Percentage contribution of each PFAS to the total HQ at both sampling sites

Figure 3 shows that the compounds that contributed most to total HQ values were PFDA, accounting for 66.6% of the total and ranging from 2.1E-02 (El Serrallo, low scenario, adults) to 3.8E-01 (El Serrallo, high scenario, infants and children); PFOA, representing 5.10% of the total and ranging from 1.3E-03 (Constantí, low scenario, adults) to 4.2E-02 (El Serrallo, high scenario, children); and PFOS, with a contribution of 28.2% to the total and ranging from 7.8E-03 (Constantí, low scenario, adults) to 2.1E-01 (Constantí, high scenario, children). The other compounds were up to three orders of magnitude lower, with HQ values ranging from 2.4E-08 (PFUnDA, El Serrallo, low scenario, adults) to 1.5E-04 (PFHxS, El Serrallo, high scenario, children). This accounted for 0.04% of the total HQ, with PFNA (0.01%), PFHxS (0.02%), and PFBA (0.007%) having the highest percentages. According to EPA guidelines (NATA 2014), an ∑HQ < 1 indicates low risk, and an ∑HQ < 0.1 represents negligible risk. In the present study, the sum of HQ values (ΣHQ) ranged from 3.1E-02 (El Serrallo, low scenario, adults) to 5.5E-01 (El Serrallo, high scenario, children). The values obtained therefore suggest a negligible risk, but when it comes to the high scenario, they indicate a low risk.

Our study shows HQ values for PFOA that are up to two orders of magnitude higher than those reported in other studies. Liu et al. (2019), for example, reported HQ values for PFOA between 4.6E-06 and 4.0E-05 and for PFOS between 1.8E-07 and 2.2E-06. Similarly, Liu et al. (2015) reported HQ values of 2.4E-07 for PFOA and 3.5E-06 for PFOS, which are significantly lower than the results in our study. However, the HQ values for other compounds in the present study, such as for PFBA (1.5E-06–3.7E-05) and PFUnDA (2.4E-08–8.0E-07), are more in line with the ranges observed in previously mentioned studies.

The CR values were obtained only for PFOA and PFOS (Table 3) since they are the only compounds with available SFo values (Table 4S). Total CR (∑CR) values ranged from 1.2E-06 (El Serrallo, low scenario, adults) to 3.7E-05 (El Serrallo, high scenario, infants), all of which were below the 10^−4^ value established by the USEPA to classify cancer risk as severe (Ma et al. 2014). According to the criteria suggested by Alani et al. (2021), the individual CR values for PFOS, which were between 3.1E-08 (Constantí, low scenario, adults) and 8.7E-07 (Constantí, high scenario, children), indicated a very low risk. In contrast, PFOA presented values ranging from 1.1E-06 (Constantí, low scenario, adults) to 3.7E-05 (El Serrallo, high scenario, children), which corresponded to a low risk.

## Conclusions

The analytical method, based on PLE/LC-MS/MS, enabled the quantification of 20 PFASs in PM_10_ samples at concentrations in the low pg m^−3^ range. Air samples were collected in two different residential areas of a coastal city with different proximities to industrial complexes, yielding similar results at both sites. The most prevalent compounds were PFBA, PFOS, and PFPA, with average values ranging from 8.05 mg m^−3^ to 106 pg m^−3^ and detection rates of 100%. PFNA was found in all the samples analyzed but at non-quantifiable levels. The remaining PFASs were detected at average values between 0.20 pg m^−3^ PFUnDA and 8.79 pg m^−3^ PFBS and lower detection rates (20% and 100%).

Non-carcinogenic (HQ) and carcinogenic risks (CR) were estimated for infants, children, and adults under a low scenario and a high scenario. Risk assessment results generally indicated negligible or low non-carcinogenic risk at the two urban sites evaluated, with total HQ values between 3.1E-02 and 5.5E-01 and PFOS, PFOA, and PFDA contributing the most. Carcinogenic risk (CR) was determined for only two compounds (PFOA and PFOS), with values ranging from 1.2E-06 to 3.7E-05, being considered a low risk.

## Supplementary Information

Below is the link to the electronic supplementary material.

ESM1 (DOCX 2.18 MB)

## Data Availability

All data supporting the findings of the present study is available from the corresponding author on reasonable request.
