# Supplementary material for: Determination of per- and polyfluoroalkyl substances in air samples from urban areas close to industrial complexes and human risk assessment
Source: Environ Sci Pollut Res Int. 2026 Jan 25;33(5):1725–37. doi: 10.1007/s11356-026-37431-6 (PMC12901172; doi:10.1007/s11356-026-37431-6)
Supplement: Supplementary file 1 — (DOCX 2.18 MB) [file 11356_2026_37431_MOESM1_ESM.docx]

**Supplementary information**

**Determination of Per- and Polyfluoroalkyl Substances in air samples from urban areas close to industrial complexes and human risk assessment**

Reyes García-Garcinuño^1^, Massimo Picardo^2^, Josepa Fabregas^2^, Laura Vallecillos^1^, Francesc Borrull^1^*, Rosa Maria Marcé^1^

^1^ Universitat Rovira i Virgili, Department of Analytical Chemistry and Organic Chemistry, Campus Sescelades, Marcel·lí Domingo, 1, Tarragona 43007, Spain

^2^ Consorci d’Aigües Tarragona, Ctra. Nacional 340, Km.1094, L’Ampolla 43895, Tarragona, Spain

^*^ e-mail: [francesc.borrull@urv.cat](mailto:francesc.borrull@urv.cat)

**TABLES**

**Table 1S.** Acronyms and full chemical names of the target compounds.

| **Compounds** | **Acronyms** |
| --- | --- |
| Perfluoro-n-butanoic acid | PFBA |
| Perfluoro-n-(^13^C_4_)butanoic acid | ^13^C PFBA |
| Perfluoro-n-pentanoic acid | PFPA |
| Perfluoro-n-(^13^C_5_)pentanoic acid | ^13^C PFPA |
| Perfluoro-n-hexanoic acid | PFHxA |
| Perfluoro-n-(1,2,3,4,6-^13^C_5_)hexanoic acid | ^13^C PFHxA |
| Sodium perfluoro-1-(2,3,4-^13^C_3_)butanesulfonate | ^13^C PFBS |
| Potassium perfluoro-1-butanesulfonate | PFBS |
| Perfluoro-n-heptanoic acid | PFHpA |
| Perfluoro-n-(1,2.3.4-^13^C_4_)heptanoic acid | ^13^C PFHpA |
| Sodium perfluoro-1-pentanesulfonate | PFPS^b^ |
| Perfluoro-n-octanoic acid | PFOA |
| Perfluoro-n-(^13^C_8_)octanoic acid | ^13^C PFOA |
| Sodium perfluoro-1-hexanesulfonate | PFHxS |
| Sodium perfluoro-1-(1,2,3-^13^C_3_)hexanesulfonate | ^13^C PFHxS |
| Perfluoro-n-nonanoic acid | PFNA |
| Perfluoro-n-(^13^C_9_)nonanoic acid | ^13^C PFNA |
| Sodium perfluoro-1-heptanesulfonate | PFHpS^b^ |
| Perfluoro-n-decanoic acid | PFDA |
| Perfluoro-n-(1,2,3,4,5,6-^13^C_6_)decanoic acid | ^13^C PFDA |
| Sodium perfluoro-1-octanesulfonate | PFOS |
| Sodium perfluoro-1-(^13^C_8_)octanesulfonate | ^13^C PFOS |
| Sodium perfluoro-1-nonanesulfonate | PFNS^a^ |
| Perfluoro-n-undecanoic acid | PFUnDA |
| Perfluoro-n-(1,2,3,4,5,6,7-^13^C_7_)undecanoic acid | ^13^C PFUnDA |
| Sodium perfluoro-1-decanesulfonate | PFDS^a^ |
| Perfluoro-n-dodecanoic acid | PFDoA |
| Perfluoro-n-(1,2-^13^C_2_)dodecanoic acid | ^13^C PFDoA |
| Sodium perfluoro-1-undecanesulfonate | PFUnS^a^ |
| Sodium perfluoro-1-dodecanesulfonate | PFDoS |
| Perfluoro-n-tridecanoic acid | PFTrA^c^ |
| Sodium perfluoro-1-tridecanesulfonate | PFTrS^d^ |
| Perfluoro-n-(1,2-^13^C_2_)tetradecanoic acid | ^13^C PFTeDA |

**Table 2S.** Acronyms, retention time (tR, min), MRM mass transitions with the collision energy, instrumental limit of detection (ILOD) and instrumental limit of quantification (ILOQ) of target and isotopically labelled PFAS standards of the target compounds determined in this study.

| **Compounds** | **t_R_ (min)** | **Transitions**  **Quantifier ion 🡪 Qualifier ion**  **(Collision energy, eV)** | | **ILOD**  **(µg L^-1^)** | **ILOQ**  **(µg L^-1^)** |
| --- | --- | --- | --- | --- | --- |
| PFBA | 6.9 | 213🡪169 (4) | - | 0.10 | 0.50 |
| ^13^C PFBA | 7.0 | 217🡪172 (4) |  |  |  |
| PFPA | 7.8 | 263🡪219 (4) | - | 0.09 | 0.50 |
| ^13^C PFPA | 7.8 | 268🡪223 (4) |  |  |  |
| PFHxA | 8.6 | 313🡪269 (4) | 313🡪119 (18) | 0.17 | 0.50 |
| ^13^C PFHxA | 8.6 | 318🡪273 (4) |  |  |  |
| ^13^C PFBS | 9.1 | 302🡪80 (40) |  |  |  |
| PFBS | 9.2 | 299🡪99 (30) | 299🡪80 (40) | 0.08 | 0.50 |
| PFHpA | 9.2 | 363🡪319 (6) | 363🡪169 (18) | 0.17 | 0.50 |
| ^13^C PFHpA | 9.3 | 367🡪322 (6) |  |  |  |
| PFPS^b^ | 9.7 | 349🡪99 (35) | 349🡪80 (39) | 0.08 | 0.50 |
| PFOA | 9.9 | 413🡪369 (6) | 431🡪169 (16) | 0.07 | 0.50 |
| ^13^C PFOA | 10.0 | 421🡪376 (6) |  |  |  |
| PFHxS | 10.3 | 399🡪119 (48) | 399🡪99 (42) | 0.08 | 0.50 |
| ^13^C PFHxS | 10.3 | 402🡪80 (42) |  |  |  |
| PFNA | 10.5 | 463🡪419 (6) | 463🡪219 (14) | 0.10 | 0.50 |
| ^13^C PFNA | 10.5 | 472🡪427 (6) |  |  |  |
| PFHpS^b^ | 10.8 | 449🡪169 (46) | 449🡪80 (50) | 0.17 | 1.0 |
| PFDA | 11.1 | 513🡪469 (8) | 513🡪219 (16) | 0.10 | 0.50 |
| ^13^C PFDA | 11.1 | 519🡪474 (8) |  |  |  |
| PFOS | 11.3 | 499🡪99 (46) | 499🡪50 (50) | 0.08 | 0.50 |
| ^13^C PFOS | 11.3 | 507🡪80 (50) |  |  |  |
| PFNS^a^ | 11.6 | 549🡪99 (56) | 549🡪80 (53) | 0.08 | 0.50 |
| PFUnDA | 11.7 | 563🡪519 (6) | 563🡪319 (15) | 0.08 | 0.50 |
| ^13^C PFUnDA | 11.7 | 570🡪525 (6) |  |  |  |
| PFDS^a^ | 12.0 | 599🡪99 (57) | 599🡪80 (54) | 0.07 | 0.50 |
| PFDoA | 12.2 | 613🡪569 (8) | 613🡪169 (26) | 0.08 | 0.50 |
| ^13^C PFDoA | 12.2 | 615🡪570 (8) |  |  |  |
| PFUnS^a^ | 12.3 | 649🡪99 (80) | 649🡪80 (120) | 0.09 | 0.50 |
| PFDoS | 12.6 | 699🡪99 (40) | 699🡪80 (100) | 0.08 | 0.50 |
| PFTrA^c^ | 12.7 | 663🡪619 (9) | 663🡪169 (86) | 0.09 | 0.50 |
| PFTrS^d^ | 13.0 | 749🡪99 (95) | 749🡪80 (200) | 0.20 | 1.0 |
| ^13^C PFTeDA | 13.1 | 715🡪670 (10) |  |  |  |
| The compounds with super index do not have analogue ISTD and the following were used for quantification: ^a^: ^13^C PFOS; ^b^: ^13^C PFHxS; ^c^: ^13^C PFDA; ^d^: ^13^C PFTeDA | | | | | |

**Table 3S.** Values used to calculate EDI (Eq 1) (Asante-Duah, 2002).

|  | **Infants**  **(1-6 years)** | **Children**  **(6-12 years)** | **Adults** |
| --- | --- | --- | --- |
| **IR (m^3·^h^-1^)** | 0.25 | 0.46 | 0.83 |
| **RR (%)** | 1 | 1 | 1 |
| **ET (h day^-1^)** | 12 | 12 | 12 |
| **EF (day year^-1^)** | 365 | 365 | 365 |
| **ED (years)** | 5 | 6 | 58 |
| **BW (kg)** | 16 | 29 | 70 |
| **AT (days)** | 1,825 | 2,190 | 21,170 |

**Table 4S.** Table with oral chronic reference dose (RfD) and oral slope factor (SfO) values for PFASs (RAIS, 2025).

| **Compounds** | **RfD (mg kg_bw_^-1^ day^-1^)** | **Source** | **SfO (mg kg_bw_^-1^ day^-1^)^-1^** | **Source** |
| --- | --- | --- | --- | --- |
| PFBA | 1.00E-03 | IRIS^a^ current |  |  |
| PFHxA | 5.00E-04 | IRIS current |  |  |
| PFBS | 3.00E-04 | PPRTV^b^ Current |  |  |
| PFOA | 3.00E-08 | DWSHA^c^ | 2.93E+04 | DWSHA |
| PFHxS | 2.00E-05 | ATSDR^d^ Final |  |  |
| PFNA | 3.00E-06 | ATSDR Final |  |  |
| PFDA | 2.00E-09 | IRIS current |  |  |
| PFOS | 1.00E-07 | DWSHA | 3.95E+01 | DWSHA |
| PFUnDA | 3.00E-04 | WI^e^ Current |  |  |
| PFDoA | 5.00E-05 | WI Current |  |  |
| ^a^Integrated Risk Information System  ^b^Provisional Peer-Reviewed Toxicity Value  ^c^Drinking Water Standards and Health Advisores  ^d^Agency for Toxic Substances and Disease Registry  ^e^Weight of Evidence | | | | |

**Table 5S.** Extraction recoveries to MeOH and ACN as a solvent extraction and temperatures of 70 ^o^C and 100 ^o^C (RSD %; n = 3).

| **Compounds** | **MeOH** | |  | **ACN** | |
| --- | --- | --- | --- | --- | --- |
|  | **70 ^o^C** | **100 ^o^C** |  | **70 ^o^C** | **100 ^o^C** |
| PFBA | 85 (5) | 84 (7) |  | 77 (1) | 73 (10) |
| PFPA | 102 (5) | 91 (6) |  | 97 (3) | 89 (10) |
| PFHxA | 113 (5) | 94 (4) |  | 107 (3) | 100 (8) |
| PFBS | 85 (5) | 87 (8) |  | 84 (6) | 79 (12) |
| PFHpA | 126 (4) | 102 (5) |  | 122 (4) | 114 (8) |
| PFPS | 79 (9) | 79 (6) |  | 70 (6) | 64 (10) |
| PFOA | 116 (5) | 96 (4) |  | 111 (5) | 104 (8) |
| PFHxS | 79 (7) | 87 (7) |  | 74 (6) | 70 (12) |
| PFNA | 97 (6) | 87 (6) |  | 92 (6) | 85 (9) |
| PFHpS | 79 (6) | 80 (9) |  | 76 (8) | 71 (11) |
| PFDA | 94 (6) | 88 (5) |  | 88 (5) | 80 (9) |
| PFOS | 74 (5) | 80 (9) |  | 73 (8) | 71 (13) |
| PFNS | 72 (5) | 77 (11) |  | 71 (9) | 67 (11) |
| PFUnDA | 81 (6) | 83 (9) |  | 76 (8) | 72 (10) |
| PFDS | 86 (4) | 85 (8) |  | 84 (8) | 77 (13) |
| PFDoA | 93 (9) | 94 (7) |  | 87 (3) | 80 (14) |
| PFUnS | 83 (1) | 90 (9) |  | 82 (8) | 74 (5) |
| PFDoS | 106 (27) | 76 (5) |  | 72 (17) | 57 (11) |
| PFTrA | 137 (29) | 94 (2) |  | 89 (17) | 70 (15) |
| PFTrS | 138 (30) | 113 (5) |  | 80 (22) | 66 (11) |

**Table 6S.** EDIs (pg kg_bw_^-1^ day^-1^) calculated for each target compound in two different exposure scenarios for three populations groups and in two sites.

| **Compounds** | **Estimated Daily Intake (pg kg_bw_^-1^ day^-1^)** | | | | | | | | | | | | | |  |
| --- | --- | --- | --- | --- | --- | --- | --- | --- | --- | --- | --- | --- | --- | --- | --- |
|  | **El Serrallo** | | | | | | | **Constantí** | | | | | | |  |
|  | **Low scenario** | | |  | **High scenario** | | | **Low scenario** | | |  | **High scenario** | | | |
|  | **Infants** | **Children** | **Adults** |  | **Infants** | **Children** | **Adults** | **Infants** | **Children** | **Adults** |  | **Infants** | **Children** | **Adults** | |
| PFBA | 4.3E+00 | 4.3E+00 | 1.5E+00 |  | 3.0E+01 | 3.1E+01 | 1.1E+01 | 9.6E+00 | 9.7E+00 | 3.4E+00 |  | 3.7E+01 | 3.7E+01 | 1.3E+01 | |
| PFOS | 2.5E+00 | 2.5E+00 | 8.9E-01 |  | 1.3E+01 | 1.3E+01 | 4.7E+00 | 2.2E+00 | 2.2E+00 | 7.8E-01 |  | 2.0E+01 | 2.1E+01 | 7.3E+00 | |
| PFPA | 4.5E-01 | 4.5E-01 | 1.6E-01 |  | 7.0E+00 | 7.1E+00 | 2.5E+00 | 2.7E+00 | 2.7E+00 | 9.6E-01 |  | 3.1E+01 | 3.1E+01 | 1.1E+01 | |
| PFBS | 3.0E-01 | 3.0E-01 | 1.1E-01 |  | 7.9E+00 | 8.0E+00 | 2.9E+00 | 1.3E-01 | 1.3E-01 | 4.7E-02 |  | 2.0E+00 | 2.0E+00 | 7.1E-01 | |
| PFTrS | 3.3E-01 | 3.3E-01 | 1.2E-01 |  | 6.5E+00 | 6.6E+00 | 2.4E+00 | 1.7E-01 | 1.7E-01 | 6.0E-02 |  | 1.3E+00 | 1.3E+00 | 4.5E-01 | |
| PFHxS | 2.4E-01 | 2.4E-01 | 8.5E-02 |  | 2.9E+00 | 2.9E+00 | 1.0E+00 | 1.4E-01 | 1.4E-01 | 4.9E-02 |  | 1.4E+00 | 1.4E+00 | 5.1E-01 | |
| PFPS | 2.5E-01 | 2.5E-01 | 8.9E-02 |  | 2.1E+00 | 2.2E+00 | 7.7E-01 | 9.5E-02 | 9.7E-02 | 3.4E-02 |  | 9.0E-01 | 9.2E-01 | 3.3E-01 | |
| PFOA | 1.1E-01 | 1.1E-01 | 4.0E-02 |  | 1.2E+00 | 1.3E+00 | 4.4E-01 | 1.1E-01 | 1.1E-01 | 3.9E-02 |  | 7.8E-01 | 8.0E-01 | 2.8E-01 | |
| PFHpS | 7.3E-02 | 7.4E-02 | 2.6E-02 |  | 6.1E-01 | 6.2E-01 | 2.2E-01 | 4.3E-02 | 4.4E-02 | 1.5E-02 |  | 3.9E-01 | 3.9E-01 | 1.4E-01 | |
| PFDoS | 4.8E-02 | 4.9E-02 | 1.7E-02 |  | 7.2E-01 | 7.3E-01 | 2.6E-01 | 4.5E-02 | 4.6E-02 | 1.6E-02 |  | 4.6E-01 | 4.6E-01 | 1.6E-01 | |
| PFHxA | 9.5E-02 | 9.6E-02 | 3.4E-02 |  | 4.3E-01 | 4.3E-01 | 1.5E-01 | 2.6E-01 | 2.7E-01 | 9.4E-02 |  | 1.6E+00 | 1.7E+00 | 5.9E-01 | |
| PFDA | 1.2E-01 | 1.2E-01 | 4.2E-02 |  | 7.5E-01 | 7.6E-01 | 2.7E-01 | 1.6E-01 | 1.6E-01 | 5.7E-02 |  | 5.7E-01 | 5.8E-01 | 2.1E-01 | |
| PFDoA | 4.0E-03 | 4.1E-03 | 1.4E-03 |  | 4.4E-01 | 4.4E-01 | 1.6E-01 | 7.5E-02 | 7.6E-02 | 2.7E-02 |  | 3.0E-01 | 3.1E-01 | 1.1E-01 | |
| PFUnS | 4.5E-02 | 4.6E-02 | 1.6E-02 |  | 5.2E-01 | 5.3E-01 | 1.9E-01 | 7.8E-02 | 7.9E-02 | 2.8E-02 |  | 1.1E+00 | 1.2E+00 | 4.1E-01 | |
| PFDS | 2.1E-02 | 2.1E-02 | 7.5E-03 |  | 3.1E-01 | 3.1E-01 | 1.1E-01 | 4.5E-02 | 4.6E-02 | 1.6E-02 |  | 5.5E-01 | 5.5E-01 | 2.0E-01 | |
| PFNS | 1.1E-02 | 1.1E-02 | 3.9E-03 |  | 5.1E-01 | 5.1E-01 | 1.8E-01 | 1.2E-02 | 1.2E-02 | 4.3E-03 |  | 2.2E-01 | 2.2E-01 | 7.8E-02 | |
| PFTrA | 2.1E-02 | 2.1E-02 | 7.6E-03 |  | 2.8E-01 | 2.8E-01 | 9.9E-02 | 4.6E-02 | 4.6E-02 | 1.6E-02 |  | 1.3E-01 | 1.4E-01 | 4.9E-02 | |
| PFUnDA | 2.0E-02 | 2.0E-02 | 7.2E-03 |  | 2.4E-01 | 2.4E-01 | 8.5E-02 | 4.1E-02 | 4.2E-02 | 1.5E-02 |  | 1.6E-01 | 1.6E-01 | 5.6E-02 | |
| PFHpA | 7.1E-02 | 7.2E-02 | 2.6E-02 |  | 4.3E-01 | 4.4E-01 | 1.6E-01 | 4.1E-02 | 4.2E-02 | 1.5E-02 |  | 1.2E-01 | 1.2E-01 | 4.3E-02 | |
| PFNA | 5.2E-02 | 5.2E-02 | 1.9E-02 |  | 8.9E-02 | 9.1E-02 | 3.2E-02 | 2.9E-02 | 2.9E-02 | 1.0E-02 |  | 8.9E-02 | 9.1E-02 | 3.2E-02 | |
| **∑PFASs** | 8.9E+00 | 9.0E+00 | 3.2E+00 |  | 7.6E+01 | 7.7E+01 | 2.8E+01 | 1.6E+01 | 1.6E+01 | 5.7E+00 |  | 1.0E+02 | 1.0E+02 | 3.6E+01 | |

**FIGURES**


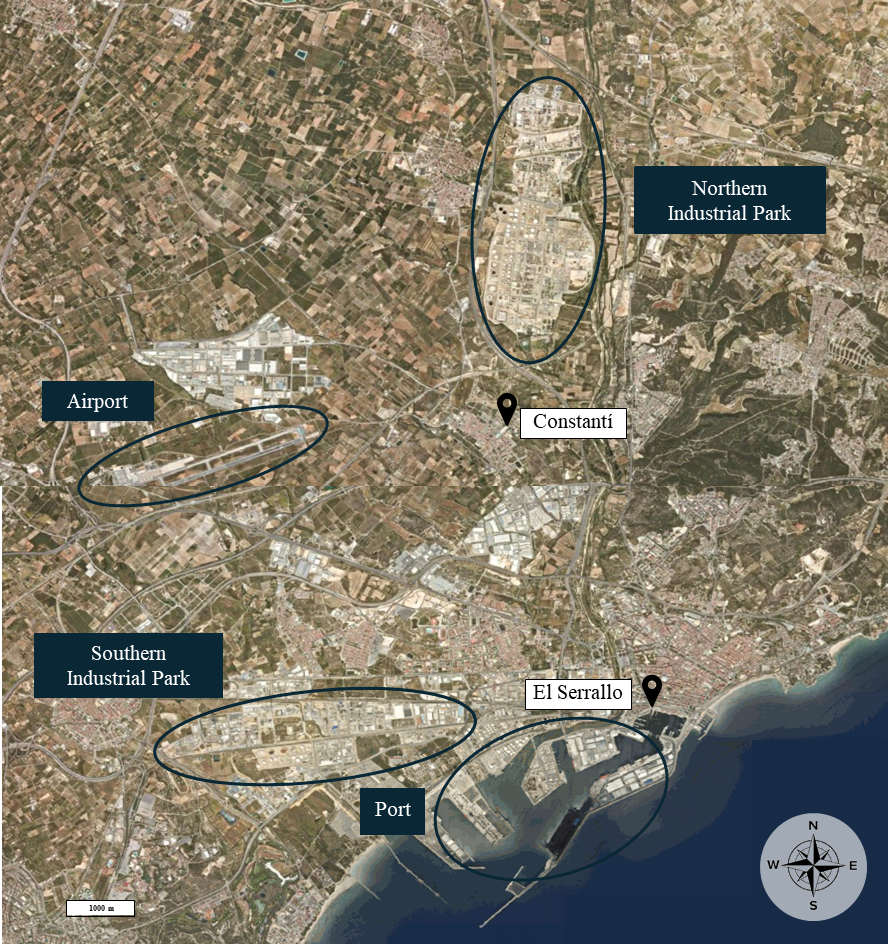


**Figure 1S.** Map of the study area showing the location of the two sampling sites (El Serrallo and Constantí), the two industrial parks in Tarragona, the port facilities, and the airport.
